# Update about “minimally verbal” children with autism spectrum disorder

**DOI:** 10.1590/1984-0462/2022/40/2020158

**Published:** 2021-09-01

**Authors:** Annio Posar, Paola Visconti

**Affiliations:** a IRCCS Istituto delle Scienze Neurologiche di Bologna, UOC Neuropsichiatria Infantile, Bologna, Italia.

**Keywords:** Autism spectrum disorder, Children, Language, Communication, Behavior, Transtorno do espectro autista, Crianças, Língua, Comunicação, Comportamento

## Abstract

**Objective:**

To review clinical and neurobiological features of minimally verbal children with autism spectrum disorder.

**Data source:**

We carried out a narrative review using the PubMed database. We considered the following search terms combined through the Boolean operator “AND”: “autism spectrum disorder”; “minimally verbal.”

**Data synthesis:**

To date, there is no shared definition of minimally verbal children with autism spectrum disorder. The heterogeneity in intellectual functioning and in linguistic abilities among these individuals suggests there is no single mechanism underlying their difficulties in learning to speak. However, the reasons why these children do not speak and the biological markers that can identify them are still unknown. Language impairment in these children can lead to several unfavorable consequences, including behavior problems (such as self-aggression, hetero-aggression, and property destruction), poorer daily living and social skills. Psychiatric comorbidities (including attention deficit/hyperactivity disorder, specific phobias, and compulsions) consist in a serious problem related to the lack of verbal language in individuals with autism spectrum disorder. Although in the literature there are very few evidence-based results, several findings suggest that an alternative and augmentative communication intervention, creating an extra-verbal communication channel, may be effective in these individuals.

**Conclusions:**

The exact definition, clinical characteristics, associated disorders, etiology, and treatment of minimally verbal subjects with autism spectrum disorder must still be further studied and understood.

## RESUMO

**Objetivo:**

Revisar as características clínicas e neurobiológicas de crianças minimamente verbais com transtorno do espectro do autismo.

**Fontes de dados:**

Realizamos uma revisão narrativa utilizando o banco de dados PubMed. Foram considerados os seguintes termos de pesquisa combinados através do operador booleano “AND”: “transtorno do espectro autista”; “Minimamente verbal”.

**Síntese dos dados:**

Até o momento, não existe uma definição compartilhada de crianças minimamente verbais com transtorno do espectro do autismo. A heterogeneidade no funcionamento intelectual e nas habilidades linguísticas entre esses indivíduos sugere que não há um mecanismo único subjacente às dificuldades de aprender a falar. No entanto, não sabemos os motivos pelos quais essas crianças não falam, nem os marcadores biológicos que podem identificá-las. O comprometimento da linguagem nessas crianças pode levar a várias conseqüências desfavoráveis, incluindo problemas de comportamento (como auto-agressão, hetero-agressão e destruição de propriedades), pior qualidade de vida diária e habilidades sociais. A comorbidade psiquiátrica (incluindo transtorno de déficit de atenção/hiperatividade, fobias específicas, compulsões) é um problema grave relacionado à falta de linguagem verbal em indivíduos com transtorno do espectro do autismo. Embora na literatura existam muito poucos resultados baseados em evidências, vários achados sugerem que uma intervenção de comunicação alternativa e aumentativa, criando um canal de comunicação extra-verbal, pode ser eficaz nesses indivíduos.

**Conclusões:**

Muito ainda precisa ser entendido sobre a definição exata, características clínicas, distúrbios associados, etiologia e tratamento de indivíduos minimamente verbais com transtorno do espectro do autismo.

## INTRODUCTION

According to the Diagnostic and Statistical Manual of Mental Disorders, Fifth Edition (DSM-5),^1^ the criteria for autism spectrum disorder (ASD) diagnosis include the presence of persisting deficits of social communication and social interaction in different contexts as well as patterns of restricted and repetitive behaviors, interests or activities. ASD symptoms often appear in early childhood, but may fully manifest later, and they significantly impact the daily functioning of affected individuals. One aspect that must not be overlooked is that symptoms are not explained by the global developmental delay or by the intellectual disability that are often associated with ASD. Compared with its previous versions,^2^ the DSM-5 proposed more specific diagnostic criteria for ASD, and language disorders are no longer considered as a central feature of ASD.^1^ However, a variable degree of verbal language impairment is usually presented by ASD individuals. At first, about 25–30% of ASD children do not develop any functional verbal language or stay minimally verbal (MV).^3^ ^–^ ^5^ That is, from an epidemiological point of view, in the general population the number of nonverbal or MV ASD children is not negligible at all. In the United States of America, ASD prevalence at the age of 8 years, according to DSM-5 criteria,^1^ is now 18.5 per 1,000 children, that is, 1 child out of 54 has an ASD.^6^ Therefore, it could be assumed that, at least in the USA, at the age of 8 years, about 5 out of 1,000 children (i.e., 1 out of 200) have an ASD and, at the same time, are nonverbal or MV. Consequently, the problem of MV children with ASD is very important also because of its considerable social and welfare costs. Nevertheless, there is no exact and broad definition of MV in the field of ASD yet; in this review, the “MV” abbreviation will also be used to refer to ASD children with completely absent speech.

## METHOD

**Figure 1:**
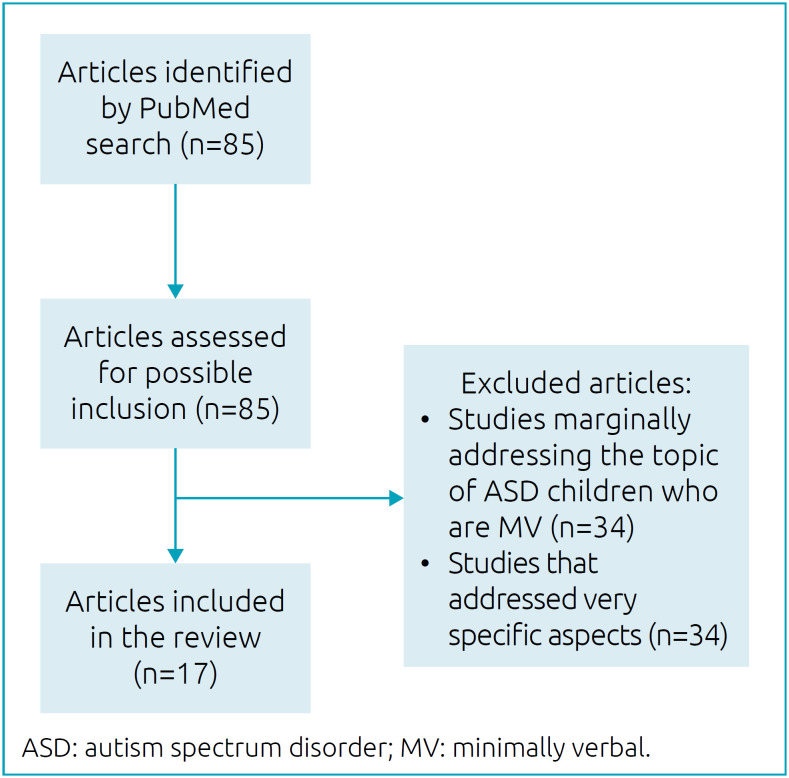
Article selection flow chart.

A narrative review using the PubMed database (United States National Library of Medicine) was carried out. The following search terms combined through the Boolean operator “AND” were considered: “autism spectrum disorder”; and “minimally verbal.” Initially, 85 studies were selected and read. Then, studies that only marginally addressed the topic of ASD children who are MV were excluded as well as those addressing very specific aspects of this topic. Both authors of the present review participated in this selection. Thus, 17 studies were finally selected, comprising reviews (either systematic or non-systematic) and original articles written in English, performed anywhere, addressing the subject of definition, diagnosis, neurobiology, clinical findings, associated problems, prognosis, and treatment of ASD individuals who are MV (Figure 1). Additional relevant bibliographic references related to the topic were also considered; such references have been mentioned in the studies selected by PubMed or have somehow been acknowledged by the authors. The references included in this review were analyzed in detail by A.P.

## RESULTS

### Language and autism spectrum disorder

The impairment of early verbal communication is usually one of the first concerns reported by parents of ASD children.^7^ In these individuals, the severity and the characteristics of language impairment greatly varies depending on the case. Strictly speaking, nonverbal children are not MV children; but these two expressions, both in clinical practice and in literature, are actually and often interchangeably used.

Most ASD children develop verbal language in the preschool period, but its progress can occur later as well.^8^ ^,^ ^9^ Their language, however, presents a series of irregularities, including in prosody, pragmatics, and semantics, which make it very peculiar, but will not be covered in this article. It is worth mentioning that the problem of communication in individuals with ASD is not limited to verbal language, but also involves other areas of communication including mimicry and gestures. Regardless of ASD, the severe or relative lack of language is a negative factor for intellectual development, as indirectly suggested several decades ago by the pioneering work of Lev Vygotskji, who studied how language acquisition can influence the cognitive development of children.^10^ However, according to Munson et al.,^11^ this does not mean that MV subjects always have a relevant deficit of nonverbal intellectual functioning, as shown by the results obtained by many of these individuals when assessed through standardized tests for intelligence quotient (IQ) bypassing the verbal channel such as the Leiter International Performance Scale.^12^ On the other hand, while some MV children with ASD have low expressive and receptive verbal skills, others have good (or relatively good) receptive abilities, which somehow seem to be related to their nonverbal skills,^13^ though being difficult to evaluate in these children.^14^ This heterogeneity in intellectual functioning and linguistic abilities between MV individuals with ASD suggests there is no single mechanism underlying their difficulties in learning to speak.^3^

Language impairment in ASD children, and particularly in MV ones, can lead to various unfavorable consequences, including behavior problems (such as self-aggression, hetero-aggression, and property destruction),^15^ ^–^ ^17^ poorer daily living and social skills.^18^ Sometimes, behavior problems can become so severe and difficult to manage, and they start being called “challenging behaviors.”^19^ As expected, these individuals are affected by negative repercussions regarding the school environment, work opportunities, and independent life, and lower quality of life and reduced opportunities for participation in the community have been reported for MV individuals.^20^ ^,^ ^21^

Psychiatric comorbidities consist in a serious problem related to the lack of verbal language in ASD individuals. Plesa Skwerer et al. studied 65 MV children and adolescents with ASD. They found a wide range of concomitant psychopathological disorders (including attention-deficit/hyperactivity disorder [ADHD], specific phobias, and compulsions) and a high degree of maladaptive behavior, not directly due to the severity of autistic symptoms, intellectual disabilities, or limitations in adaptive functioning.^22^

Williams et al. have a different perspective; the authors compared individuals with ASD hospitalized in a psychiatric institution (age range: 4–20 years): 169 MV subjects versus 177 individuals with fluent verbal language. They found no significant differences in the severity of self-injury, stereotyped behavior, and irritability (including aggression and anger) when data were controlled for age and nonverbal IQ. However, a problem of sample selection bias can be hypothesized (of which the authors were also aware): all cases were “psychiatric inpatients,” thus they were likely predisposed to behavioral problems.^23^

Concerning the prognosis of MV children with ASD, it is known that if a child manages to acquire verbal language, this usually occurs within 5 years of life.^3^ However, exceptions to this general rule have been reported, considering that verbal language can be acquired between 5 and 7 years of age or (although more rarely) even later, up to the age of 13 years.^24^ Regarding other aspects of the prognosis, the development of useful speech within the age of 5 years seems to be a very important predictive factor of better outcomes in the following years, as it concerns adaptive and social functioning.^3^

### How to define minimally verbal children?

The lack of a broad definition of MV children is somehow noteworthy. In the context of children with ASD, the proportion of those who are MV depends on the criteria used to identify them. For example, Kasari et al. defined MV children as those communicatively using “[…] a very small repertoire of spoken words or fixed phrases…”; the number of spoken words may greatly vary, ranging from 0 to 20–30, depending on several factors such as age and previous interventions.^25^ In order to identify MV children, many researchers utilize clues deriving from the diagnostic tools for ASD. For instance, according to many authors, children are considered MV if they have been assessed through the Autism Diagnostic Observation Schedule (ADOS) — Module 1.^26^ The ADOS, currently available in its second edition, is nowadays considered the gold standard for the ASD diagnosis, and the Module 1 of this scale is focused on children aged over 30 months who speak few or no words.^27^ This method may be preferable in some respects, considering that ADOS also allows the direct assessment of the individual, but it is not a foolproof method. In fact, Module 1 of the current version of ADOS consists of two submodules, devoted to children with greater or lesser language impairment, respectively; consequently, the language of children who undergo this module can widely vary from the complete absence of speech to the regular use of expressions composed of two or more words (see item A1 of ADOS Module 1).^27^ Therefore, including all these ASD children under one definition of MV could be misleading; and it is a fact that, within the definition of MV, there are very different situations.^26^

Another method is based on the use of words according to parents’ reports: it identifies children as MV when their language skills are not exceeding those of a baby aged 18 months, who mainly uses single words or gestures for communicating; this is perhaps a less objective method than the previous one, but it gives information on the real everyday life of the child. According to the results obtained in a study on a large sample including 1478 ASD individuals aged 5-18 years, through the method of ADOS Module 1, 28% of ASD children should be MV; instead, according to the method of language skills not exceeding those of a 18-month baby, only 13% should be MV.^28^

Therefore, it is evident that issues related to an exact definition of MV are paramount. For instance, in a review addressing a certain ASD intervention, grouping, within the MV phenotype, the data on the evolution of children who, at the beginning of the intervention, speak only one or two words with those of children who speak a few dozen words may lead to significant misinterpretations of the obtained results.^21^

Koegel et al. carried out a systematic review of the definitions of nonverbal or MV individuals and the communication assessment measures in intervention studies aimed at improving expressive verbal communication in ASD children. They found relatively few studies focusing on verbal expressive communication in nonverbal or MV children with ASD. The authors verified great inconsistencies in the adopted measures, in the definitions of “nonverbal” and “MV” children, and in the studied ages, which can cause confusion in the interpretation of the results of the various studies. They suggested guidelines for creating a more homogeneous evaluation protocol with systematic descriptions of the samples, in order to understand the heterogeneity in these ASD individuals and to replicate the results of the research that concerns them. The recommendations included in these guidelines refer, among other things, to the importance of: identifying the participants as nonverbal or MV; evaluating the language through standardized and observational measures, also considering a natural sample of interactive communication, if possible with a communication partner who is familiar to the child; and also examining the intellectual functioning of the child.^21^

### Neurobiology of minimally verbal individuals

Neurophysiological techniques, as well as structural and functional neuroimaging ones, have been applied to look for possible abnormalities in children with ASD, in order to explain the evolution of language.^20^ Next, only some of the most relevant obtained results are demonstrated.

Ortiz-Mantilla et al. performed an electroencephalogram-based study to investigate neural mechanisms that underlie the visual processing of common objects in MV children with ASD. A paradigm consisting of a picture, followed by a word that could be right or wrong, was presented to 10 MV children of 4–7 years old with ASD and to 15 sex- and age-matched controls. Event-related cortical responses during visual stimulus processing were studied. When compared with controls, responses of lower amplitude and longer latency were recorded in MV children with ASD as well as other bioelectric alterations in the occipital and frontal regions. The authors concluded that visual processing, both in the early and late stages, is impaired in MV children with ASD, and at least some of their linguistic alterations could derive from an impairment of cortical representations of the object and its verbal label and also from a reduced allocation of attention to visual stimuli, which would have a negative impact on lexicon acquisition.^29^

Concerning neuroimaging techniques, Knaus et al. studied possible anatomical differences between ASD individuals with more or less severely impaired expressive language and ASD individuals without such deficits using volumetric brain magnetic resonance imaging (MRI). They included 34 ASD boys aged 7–11 years divided into two groups: individuals with impaired expressive language (17 cases) and those with average or high language (17 cases) respectively. The first group was further subdivided into a low (9 cases) and an extremely low (8 cases) language subgroup (this latter basically corresponding to MV children). The volume of the planum temporale (PT) was smaller in individuals with expressive language impairment than in those without it. The right PT volume was positively correlated with expressive, receptive, and total language abilities. The volume of the left PT was smaller in the individuals with extremely low language than in those with average or moderately low language. The authors concluded that, in ASD individuals, smaller PT volumes are associated with severe language impairments.^30^ Moreover, cortical structural abnormalities in the inferior frontal gyrus have been reported for these children.^31^

On the other hand, it has been suggested that microstructural alterations of white matter tracts, such as the arcuate fasciculus (connecting Broca’s and Wernicke’s areas) and the frontal aslant tract (connecting the posterior inferior frontal gyrus and the brain frontomesial region), showed by magnetic resonance with diffusion tensor imaging (DTI), may be involved in the impairment of spoken language of MV subjects with ASD.^32^

While ASD individuals often present a more or less severe impairment of language, music skills are usually preserved, although brain regions involved in these functions commonly overlap. Based on this observation, Lai et al. found a higher activation in the left inferior frontal gyrus in response to song stimulation through functional MRI in 36 ASD low-functioning children, who were mostly MV, but a lower activation in response to speech stimulation. These results support the hypothesis of an atypical attention to auditory stimuli in ASD individuals: reduced for speech, but increased for songs.^33^

However, despite the described findings, it is still unknown how the reported brain functional and structural alterations might explain the language problems in ASD children.^20^ To date, to the best of the authors’ knowledge, there are no biological markers that identify MV individuals with ASD.

### Cognitive mechanisms and language in autism spectrum disorder

Several studies have addressed the topic of cognitive mechanisms likely involved in the altered or even absent development of verbal language in ASD children. Although these studies have not achieved unequivocal and conclusive results, they suggested the presence of data supporting the involvement of some variables related to social communication in expressive language development, such as joint attention, spontaneous play, gestures (including pointing), and imitation (both vocal and motor) abilities; moreover, the early nonverbal intellectual ability has been related to the development of expressive language.^34^ ^–^ ^37^ In particular, joint attention is the skill (commonly developed in the first year of life) of responding to others’ social initiatives and spontaneously engaging in social interactions with other people, in addition to integrating these two abilities.^38^ Joint attention has been found to predict the development of language skills in both typically-developing children and those with ASD^34^ ^,^ ^38^, and this is evidently a very important finding also regarding interventions aimed at the development of long-term spoken language.^39^

Thus, an impairment of joint attention, pointing, spontaneous play, imitation skills, together with a more or less severe delay in verbal language development, are likely to be among the early symptoms of autism most often reported by parents when the child ages up to 18 months old.^40^

### Treatment for minimally verbal children with autism spectrum disorder

Considering that most studies investigated communication-focused interventions in verbal children with ASD, while their efficacy in MV children with ASD is unknown, Brignell et al. performed a systematic review on this last issue.^20^ They considered randomized controlled trials (RCTs) related to communication-focused interventions for children with ASD who were MV, that is, according to their definition, children who spoke less than 30 functional words and/or were unable to use verbal language alone to communicate. The authors found only two RCTs that compared MV children undergoing communication-focused interventions with MV controls undergoing their usual treatment. One RCT^41^ used a verbally-based intervention performed by parents at home (focused playtime intervention — FPI), while the other one^42^ used an alternative and augmentative communication (AAC) intervention carried out by teachers at school through the Picture Exchange Communication System (PECS), which allows individuals to quickly communicate to others, for example, their needs using picture cards. The FPI study considered 70 MV children with ASD and focused on promoting coordinated toy play between the parent (who had received a specific training) and the child. The AAC study considered 84 MV children with ASD who were not using the PECS method yet. Brignell et al. found a very low overall quality of evidence due to the risk of bias (including the lack of blinding for participants and personnel), imprecision (small size of the samples), and also because only one trial was identified for each intervention type (verbally-based and AAC, respectively). Both trials mainly focused on verbal and nonverbal communication outcomes, and the study concerning AAC intervention also focused on social communication skills. The FPI trial showed no significant results in verbal communication. Nevertheless, cases with poorer expressive language before treatment improved more than those with better expressive language, and FPI promoted expressive language improvements in some cases.^41^ The AAC study found that cases enrolled immediately postintervention used significantly more verbal initiations and PECS, but unfortunately these gains were not confirmed 10 months later. In immediately postintervention, no significant improvements were found with AAC on speech frequency, verbal expressive vocabulary, or social communication skills or pragmatic language.^42^ Overall, in most cases, both FPI and AAC trials showed no sustained gains in verbal or nonverbal communication skills.^20^

Assuming that the role of parent education is essential for the treatment of ASD subjects and in particular of MV ones, Koegel et al. performed a systematic review on the procedures of parent education in intervention studies aimed at improving verbal communication of MV children. Unfortunately, they found that only 36 studies have considered a parent education component; in most articles, parent education programs were not clearly described, and only in few studies the implementation of parents’ intervention was scored.^43^

**Figure 2:**
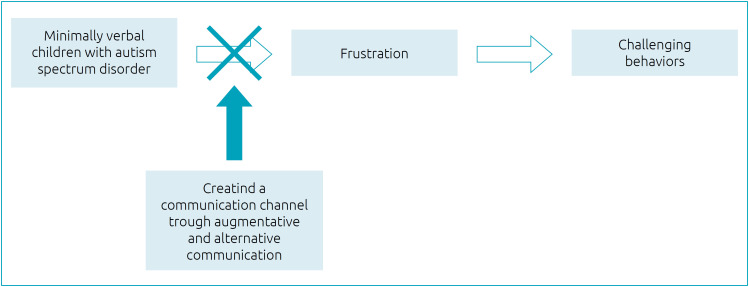
Augmentative and alternative communication in minimally verbal children with autism spectrum disorder. Each white arrow indicates a predisposing effect; the black arrow indicates an inhibiting effect. See text for details.

However, although the literature on the matter has provided very few evidence-based results, several findings suggest that an intervention, such as the AAC, considering a communication channel for the individual, may be effective in stopping the sequence of events that, in MV children with ASD, can cause frustration to build up and trigger challenging behaviors and, finally, accentuate social isolation (Figure 2). In particular, PECS represents an effective means of enabling functional communication for individuals who have little or no speech, even though it showed a relative lack of relevant gains concerning speech.^44^ ^–^ ^47^ It is worth noting that AAC includes, in addition to PECS, other types of interventions, including the use of speech-generating devices (SGDs) that, upon command of the user, are able to produce previously recorded or computer-generated speech.^48^ SGDs seem to be most effective for ASD individuals without intellectual/developmental disorders (IDD), especially in preschool age, whereas PECS seems to be most effective for ASD individuals with IDD.^48^

## DISCUSSION

First, the limitations of this narrative review should be mentioned, which are basically related to a possible selection bias due to the subjective evaluation of the studies on the part of the authors. However, it is worth highlighting that the study methodology of a narrative review is more indicated than a systematic review in cases when the aim is to provide a broad perspective on a subject.^49^

Although to date there are many notions about the clinical aspects of ASD, including diagnostic criteria, associated disorders, prognosis, and effective treatments, there is no agreement yet on the exact and detailed definition of MV children with ASD or on how to precisely identify them. Hence, the exact number of MV children with ASD is unknown, but it is surely high, particularly based on the most recent epidemiological data about the general prevalence of ASD.^6^ From the authors’ perspective, in order to better understand the clinical features and the neurobiological correlates of this subgroup of individuals with ASD, it would be appropriate to define them with very rigorous diagnostic criteria, considering individuals as MV only when their language level corresponds, at most, to the level of single words, and their chronological age is at least 5–7 years, considering that a significant recovery of language skills is much more unlikely later in life.^24^ It is evident, for example, that the situation of a 3-year-old child who does not speak, still theoretically being susceptible to great improvements, could be very different in prognostic terms from that of a 10-year-old child who does not speak and who probably never will in his/her life. Perhaps also due to these uncertainties regarding a correct definition, the reasons why MV children with ASD do not speak are unknown, although some hypotheses emerged from the results of studies based on neurophysiological and structural/functional neuroimaging data. At a clinical level, in the context of children with ASD, the authors believe that the effort to find any correlate in terms of neuroimaging should be primarily made precisely in those who are MV, seeking — for example — any alterations of the brain connectivity through DTI techniques. Furthermore, behavioral problems are more frequent and complex in MV children than in those who are able to develop language, often involving significant management difficulties, but there is lack of evidence-based demonstration of the effectiveness of ASD interventions focused on communication in MV children. Therefore, further studies must be conducted on these individuals who, not surprisingly, have been called some years ago by some authors as “the neglected end of the spectrum.”^3^

Although evidence-based data are lacking, several findings suggest that interventions, such as the AAC, and in particular PECS, may be effective in enabling functional communication in MV children with ASD.^44^ ^–^ ^47^ This type of intervention is very important because, by bypassing the lack of verbal language, it can give a means of communication to these individuals who, among people with ASD, are certainly those who require more care and attention and whose management involves high social and welfare costs, regardless of the emotional suffering produced in their caregivers.

In addition, more research is needed to understand the natural history of MV individuals with ASD from the beginning. For example, is it possible that subjects who display developmental regression as their onset mode (the so-called regressive autism) are, therefore, (at least for the most part) the same who remain MV during their life? Still nowadays, it is very difficult to find a clear and conclusive answer to a relatively simple question such as this in the literature.^4^

In conclusion, the authors believe that the issue of MV individuals with ASD should be addressed by finding a shared definition of this condition characterized by very rigorous clinical criteria and by systematically studying these patients at a genetic, neurophysiological, and neuroimaging levels, also aiming at finding any distinctive neurobiological correlates that may possibly represent the starting point for more targeted and specific enabling interventions.
